# Group A *Streptococcus emm* Gene Types in Pharyngeal Isolates, Ontario, Canada, 2002–2010

**DOI:** 10.3201/eid1711.110159

**Published:** 2011-11

**Authors:** Patrick R. Shea, Amy L. Ewbank, Javier H. Gonzalez-Lugo, Alexandro J. Martagon-Rosado, Juan C. Martinez-Gutierrez, Hina A. Rehman, Monica Serrano-Gonzalez, Nahuel Fittipaldi, Stephen B. Beres, Anthony R. Flores, Donald E. Low, Barbara M. Willey, James M. Musser

**Affiliations:** The Methodist Hospital Research Institute, Houston, Texas, USA (P.R. Shea, A.L. Ewbank, J.H. Gonzalez-Lugo, A.J. Martagon-Rosado, J.C. Martinez-Gutierrez, H.A. Rehman, M. Serrano-Gonzalez, N. Fittipaldi, S.B. Beres, A.R. Flores, J.M. Musser); Texas Children’s Hospital, Houston (A.R. Flores); Baylor College of Medicine, Houston (A.R. Flores); Mount Sinai Hospital/University Health Network, Toronto, Ontario, Canada (D.E. Low, B.M. Willey); Ontario Agency for Health Protection and Promotion, Toronto (D.E. Low); University of Toronto, Toronto (D.E. Low)

**Keywords:** Streptococcus pyogenes, GAS, pharyngitis, group A streptococcus, Canada, emm, bacteria

## Abstract

Group A *Streptococcus* (GAS) is a human-adapted pathogen that causes a variety of diseases, including pharyngitis and invasive infections. GAS strains are categorized by variation in the nucleotide sequence of the gene (*emm*) that encodes the M protein. To identify the *emm* types of GAS strains causing pharyngitis in Ontario, Canada, we sequenced the hypervariable region of the *emm* gene in 4,635 pharyngeal GAS isolates collected during 2002–2010. The most prevalent *emm* types varied little from year to year. In contrast, fine-scale geographic analysis identified inter-site variability in the most common *emm* types. Additionally, we observed fluctuations in yearly frequency of *emm3* strains from pharyngitis patients that coincided with peaks of *emm3* invasive infections. We also discovered a striking increase in frequency of *emm89* strains among isolates from patients with pharyngitis and invasive disease. These findings about the epidemiology of GAS are potentially useful for vaccine research.

## Abstract 2

Determination of *emm* variations may help improve vaccine design.

Group A *Streptococcus* (GAS) is a gram-positive bacterial pathogen responsible for ≈600 million cases of pharyngitis each year worldwide (*1*). The widespread prevalence of this disease results in considerable costs, estimated to exceed $200 million annually in the United States alone (*2*). In addition to acute pharyngitis, GAS causes several other human diseases, ranging from relatively mild to more severe, such as necrotizing fasciitis, soft tissue infections, glomerulonephritis, acute rheumatic fever, and streptococcal toxic shock syndrome. Thus, infections caused by GAS are a major public health concern in the United States and Canada and throughout the world.

GAS strains are classified mainly on the basis of variation in a cell-surface molecule known as M protein, encoded by the *emm* gene (*3**,**4*). M protein is a critical virulence factor and a major site of the human antibody response against GAS. M type–specific immunity develops in persons recovering from some GAS infections (*5**,**6*). As a result, the portion of the *emm* gene that encodes the amino-terminal 100 residues of M protein is under strong diversifying selection pressure, and this region is hypervariable in terms of GAS types (*7*). Currently, >120 distinct *emm* types of GAS are recognized.

Despite the considerable diversity of *emm* types of GAS isolates, epidemiologic studies have found that relatively few *emm* types tend to predominate within a local population; most isolates are composed of a small number of *emm* types (*8**,**9*). In distinct geographic areas, the predominant *emm* types often vary in frequency from year to year for reasons not fully understood. In addition, sizeable outbreaks can be caused by strains of a single *emm* type or of a small number of *emm* types. Overall, this combination of factors results in a complex epidemiologic situation for GAS pharyngitis.

Recently, vaccine candidates have been identified in an effort to reduce the prevalence of GAS disease and the number of human deaths it causes (*10*). Some of these experimental vaccines are based on the amino-terminus of M protein because of the type-specific immunity that may develop after GAS infection. A multivalent vaccine has been developed that exploits the amino-terminus of the M protein from many different *emm* types (*11*). In principle, the effectiveness of this type of M-protein vaccine may be highly dependent on how well the M proteins selected for the vaccine match the *emm* types of locally circulating strains. Thus, a more complete understanding of geographic and temporal variation in *emm* type may be useful for vaccine design. Furthermore, the emergence of new variants of known M types has been documented. Knowledge of the rate and patterns of emergence of distinct *emm* types and their alleles may be critical for understanding how GAS may “escape” the immune response generated by a vaccine based on the amino-terminus of M protein.

We investigated the distribution of GAS *emm* types causing pharyngitis in Toronto, Ontario, Canada, during 2002–2010. We also examined the temporal change in *emm* types in pharyngitis cases and compared this distribution with data from a comprehensive population-based study of GAS *emm* types that were causing invasive infections in Ontario. Finally, we studied the *emm* types causing pharyngitis in multiple geographic locations across the province of Ontario in 2009 and 2010.

## Materials and Methods

### Collection of Isolates

Isolates collected from throat specimens of patients with acute pharyngitis were identified as GAS from primary media by a variety of standard methods. These GAS isolates (hereafter also referred to as pharyngeal isolates) were collected from 2002 through 2010 from multiple Ontario laboratories. GAS isolates, stripped of patient identifiers, were forwarded to Mount Sinai Hospital in Toronto for confirmation of identity and shipped to The Methodist Hospital Research Institute in Houston, Texas, for *emm* gene typing. Basic demographic information, including location where collected, age and sex of patient, and specimen collection date, was provided for isolates.

#### Toronto Isolates

During the summers (May through September) of 2002–2003 and 2006–2010, ≈500 consecutive isolates were collected each year from LifeLabs (formerly MDS), a large, centralized, commercial laboratory on the outskirts of Toronto. This facility primarily serves family medicine practices and outpatient clinics within the greater Toronto area but also acts as a catchment conduit from the surrounding region. The following number of isolates was obtained from this site per collection year: 523 (2002), 619 (2003), 502 (2006), 510 (2007), 522 (2008), 481 (2009), and 487 (2010). An additional ≈520 consecutive throat specimen GAS isolates from the Mount Sinai Hospital/University Health Network Clinical Microbiology Laboratory were collected during January 2008–March 2010.

#### Geographically Diverse Ontario Strains

Additional isolates from outlying locations of LifeLabs and Gamma Dynacare laboratory chains in London, Sudbury, and Thunder Bay, Ontario, each provided 100 consecutive GAS isolates per center during July–September 2009. The Gamma Dynacare Ottawa laboratory provided consecutive isolates up to 100 per month from July 2009 through July 2010, for a total 659 isolates. The Gamma Dynacare London branch provided 219 isolates from July through September 2009, and the distantly located North Bay and Elliot Lake branches together provided 36 GAS isolates from July through October 2009.

### *emm* Type Assignment

GAS isolates were grown overnight at 37°C with 5% CO_2_ on trypticase soy agar plates containing 5% sheep blood (TSAII; Becton Dickinson, Franklin Lakes, NJ, USA). Genomic DNA was obtained by boiling a sample obtained by streaking from multiple colonies in 0.05 mol/L NaOH for 2 min. The crude cell lysates were centrifuged for 2 min at 2,000 × *g*, and 2 μL of the lysate was used in PCRs. The hypervariable region of the *emm* gene that encodes the amino-terminus of M protein was amplified by PCR by using primers emm1 5′-TATT(C/G)GCTTAGAAAATTAA-3′ and emm2 5′-GCAAGTTCTTCAGCTTGTTT-3′. PCR products were purified by using 96-well ultrafiltration plates (EdgeBio, Gaithersburg, MD, USA), according to the manufacturer’s instructions; products were suspended in 100 μL distilled water. Cycle sequencing was performed with the Big Dye version 3.1 dye-terminator kit (Applied Biosystems, Foster City, CA, USA) by using primer emm1. Unincorporated fluorescent dye terminators were removed with 96-well gel-filtration cartridges (EdgeBio). Sequencing reactions were analyzed with a 3730xl DNA sequencer (Applied Biosystems), and chromatograms were analyzed with Sequencher version 4.9 (GeneCodes, Ann Arbor, MI, USA). High quality sequences were trimmed to 220 nt in length and compared with reference sequences in the Centers for Disease Control and Prevention (CDC) *emm* database (ftp://ftp.cdc.gov/pub/infectious_diseases/biotech/tsemm) by using the BLAST algorithm (http://blast.ncbi.nlm.nih.gov/Blast.cgi). Data analysis and graphing were performed with the GraphPad software package (Prism, La Jolla, CA, USA). The invasive index of each *emm* type was calculated by dividing its frequency in invasive infections by frequency in pharyngitis infections.

## Results

### Overview of Pharyngitis Strains

We determined the *emm* type for 4,635 GAS isolates that were causing acute pharyngitis in the province of Ontario during 2002–2010. Of these, 3,209 isolates were collected from the greater Toronto metropolitan region, and 1,426 isolates were obtained from 5 sites located throughout Ontario (London, Ottawa, North Bay/Elliot Lake, Sudbury, and Thunder Bay). The mean age of patients was 16.1 years (range 8 months to 105 years).

### Distribution of *emm* Types in Toronto GAS Pharyngitis Strains

**Figure 1:**
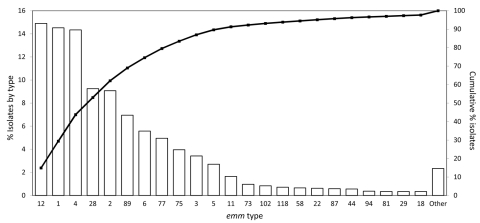
Distribution of group A *Streptococcus* (GAS) *emm* types collected in Toronto, Ontario, Canada, 2002–2010. Thirty-four GAS *emm* types with <10 isolates each (≈0.3% of total) comprise the “other” category. Line graph showing cumulative percentage is superimposed with percentage scale shown on right.

**Figure 2:**
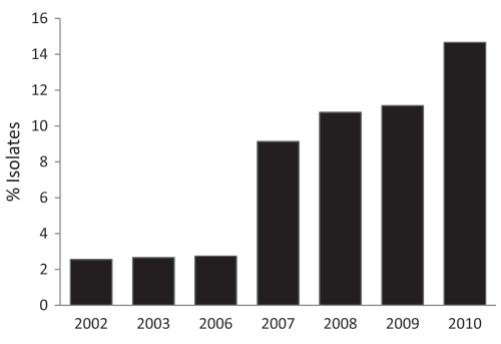
Annual frequency of *emm*89 isolates among patients with group A *Streptococcus* pharyngitis, Toronto, Ontario, Canada, 2002–2010.

Consistent with findings from previous surveys of GAS isolates that have caused pharyngitis in North America, Europe, and elsewhere (*8**,**9**,**12*), we found that a relatively small number of *emm* types dominated. For example, the 6 most prevalent *emm* types collected in Toronto during 2002–2010 were (in order of prevalence) *emm*12, *emm*1, *emm*4, *emm*28, *emm*2*,* and *emm*89 (Figure 1). These 6 *emm* types came from 68.9% of the pharyngeal isolates, whereas 29 *emm* types came from the remaining 31.1% of the isolates. Analysis of the annual change in *emm* type distribution indicated that, with few exceptions, these 6 types were consistently the most commonly collected. This finding suggests that the *emm* type population dynamic is relatively stable. However, *emm*89 strains were a key exception. The frequency of *emm*89 isolates increased 5-fold over the study period, increasing from 2.6% of isolates in 2002 to 14.7% in 2010 (Figure 2). In 2010, *emm*89 isolates were the second most common *emm* type among pharyngitis specimens in our sample. These data indicate a recent major expansion of type *emm89* strains among isolates causing pharyngitis in Toronto.

### Identification of New *emm* Alleles

We identified 20 allelic variants of 8 GAS *emm* types that had not been previously described: *emm*1 (4 alleles), *emm*3 (3 alleles), *emm*5 (3 alleles), *emm*6 (4 alleles), *emm*8 (1 allele), *emm*11 (2 alleles), *emm*12 (2 alleles), and st106M (1 allele). Nucleotide sequences for these alleles have been submitted to the CDC *Streptococcus pyogenes* *emm* sequence database (designations listed in Table 1). Seventeen of these alleles differed from the most closely related reference sequence by 1 single-nucleotide polymorphism (SNP); 2 allelic variants differed by 2 SNPs; and 1 isolate had a 6-bp in-frame insertion, resulting in the addition of 2 amino acid residues. Of the 21 SNPs identified, all but 1 resulted in a predicted amino acid substitution in the translated M-protein sequence. This excess of nonsynonymous mutations underscores the effect of the strong diversifying selection pressure acting on the *emm* gene.

**Table 1:** Newly identified *emm* allelic variants from group A *Streptococcus* pharyngeal isolates, Ontario, Canada, 2002–2010

*emm* type	Allele designation
*emm*1	*emm*1.56, *emm*1.57, *emm*1.58, *emm*1.59
*emm*3	*emm*3.63, *emm*3.64, *emm*3.65
*emm*5	*emm*5.83, *emm*5.84, *emm*5.87
*emm*6	*emm*6.76, *emm*6.77, *emm*6.78, *emm*6.79
*emm*8	*emm*8.2
*emm*11	*emm*11.10, *emm*11.11
*emm*12	*emm*12.54, *emm*12.55
st106M	st106M.5

### Ontario *emm* Types in Relation to an Experimental 26-Valent GAS Vaccine

Overall, 18 of 57 *emm* types found in the Toronto pharyngeal isolates are represented in an experimental 26-valent GAS vaccine described elsewhere (*11*). These 18 *emm* types included 11 of the 12 most prevalent *emm* types that represent strains causing 78.5% of the pharyngitis cases we studied, a number similar to estimates for the US population (*11*). Notably, the single most commonly observed GAS *emm* type (*emm*4) not included in the 26-valent experimental vaccine was very common in Toronto. For example, during 2002–2010, *emm*4 was the third most common *emm* type causing pharyngitis, and in 2007 and 2008, it was the most common *emm* type. This finding is not entirely unexpected because *emm*4 has been one of the most common serotypes identified by other pharyngitis surveys (*8**,**9**,**12**,**13*).

Although a multivalent GAS vaccine based on the amino-terminus of M protein has theoretical promise, a potential concern is the detrimental effects of allelic variation on vaccine efficacy. Virtually all new and previously described *emm* alleles collected from pharyngitis patients in Toronto contained nucleotide changes that resulted in changes in amino acid sequence, with few alleles defined only by silent nuclear polymorphisms. New *emm* alleles generated by strong diversifying selection pressure acting on the *emm* gene could provide the means by which GAS strains evade a vaccine that includes only a single variant of each M-protein serotype; that is, creating vaccine-escape mutants.

To determine how common allelic variation was in the Toronto GAS population, we examined the number of alleles for each *emm* type found in specimens from pharyngitis patients in Toronto. For the top 10 serotypes, the most prevalent allele was found in 87.2% of the isolates (range 100%–42.7%). Notably, several prevalent *emm* types had an extensive number of alleles that would encode variant M proteins. For example, of the 6 most common serotypes, 4 had >6 allelic variants, and the most common *emm* type (*emm*12) had 16 different alleles.

### Comparison of *emm* Type Distribution in Pharyngitis and Invasive GAS isolates

Previous studies have identified nonrandom associations between specific *emm* types and an increased risk for invasive infection (*14**–**17*) or increased severity of invasive infection (*18**,**19*). Thus, we tested the hypothesis that certain *emm* types were more prevalent in invasive disease isolates than in pharyngitis isolates in the Toronto region. Consistent with previous reports (*9**,**20**,**21*), we found that *emm*1 and *emm*3 strains each had an invasive index >1.0 (Table 2), which suggests that these *emm* types are overrepresented among invasive infections. Additionally, *emm*49 strains had an exceptionally high invasive index (16.7), largely because of the rarity of these strains among the pharyngeal isolates.

**Table 2:** Invasive indexes for *emm* types in group A *Streptococcus* isolates from patients with invasive disease and pharyngitis, Ontario, Canada, 2002–2010

*emm* type	Invasive disease frequency	Pharyngitis frequency	Invasive index
1	0.298	0.142	2.09
2	0.021	0.094	0.22
3	0.051	0.037	1.38
4	0.057	0.150	0.38
5	0.028	0.025	1.12
6	0.029	0.060	0.48
11	0.031	0.017	1.82
12	0.081	0.138	0.59
22	0.005	0.007	0.71
28	0.051	0.098	0.52
49	0.036	0.002	16.7
75	0.022	0.034	0.65
77	0.024	0.058	0.41
78	0.006	0.002	3.0
89	0.058	0.058	1.0
Others	0.202	0.078	2.59

**Figure 3:**
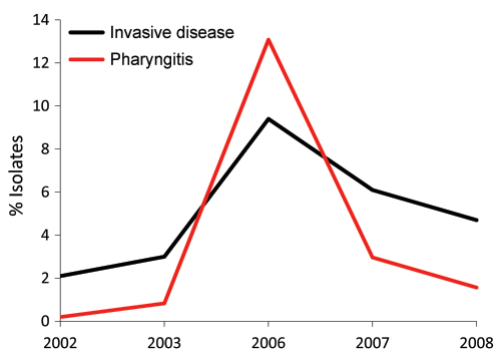
Frequency of *emm*3 strains among patients with group A *Streptococcus* pharyngitis and invasive disease, Ontario, Canada, 2002–2010, excluding 2004–2005. Black line indicates yearly frequency of invasive *emm*3 isolates; red line indicates *emm*3 frequency among pharyngeal isolates.

Comparison of the annual change in *emm* type frequencies in pharyngitis and invasive disease isolates indicated that certain *emm* types had highly variable frequencies, consistent with epidemic behavior. In particular, *emm*3 pharyngitis strains peaked in frequency in 2006 to become the fourth most common *emm* type that year. This timing corresponds with the observed peak in cases of invasive disease caused by *emm*3 strains in 2006 (Figure 3), which suggests a relationship between abundance of pharyngitis cases and invasive infections.

### Variability in Frequency Distribution of *emm* Types from Diverse Localities

Several studies have reported that *emm* type distribution can vary geographically. Generally, however, these comparisons involved localities separated by large distances. To test the hypothesis that *emm* type distribution varied over a relatively small geographic distance, we analyzed GAS isolates from pharyngitis patients at 5 additional areas across Ontario (London, Ottawa, North Bay/Elliot Lake, Sudbury, and Thunder Bay) during 2009–2010. In the aggregate, the *emm* types from the 5 geographically distinct collection sites closely resembled those found in Toronto in 2009 and 2010. The same 6 most prevalent *emm* types were found in 2009, and only 1 *emm* type differed in frequency in 2010. In general, *emm* types from individual collection sites were consistent from year to year (Table 3). However, we discovered striking inter-site variability in the distribution of *emm* types. For example, in 2009, *emm*89 was the most common *emm* type identified at 4 of the 6 localities, but *emm*89 strains were not among the 6 most common *emm* types found in Ottawa. Additionally, only 2 *emm* types were shared among the 5 most common organisms collected in North Bay and Sudbury, an unexpected result (p = 0.0007; Fisher exact test), given that these locations are separated by only ≈120 km.

**Table 3:** Five most common M types of group A *Streptococcus* from pharyngeal isolates, by location, Ontario, Canada, 2009–2010*

Year	London	Ottawa	North Bay/ Elliot Lake	Sudbury	Thunder Bay
2009	M89	M4	M89	M89	M28
	M4	M28	M75	M2	M12
	M2	M1	M4	M11	M89
	M1	M3	M1	M12	M6
	M12	M12	M77	M75	M58
2010	NA	M1	M89	NA	M28
		M3	M1		M59
		M4	M118		M1
		M28	M4		M12
		M12	M75		M89

*NA, not available.

We also observed apparent local outbreaks of certain *emm* types in some locations. In 2009 and 2010, *emm*3 strains were among the 6 most prevalent *emm* types in Ottawa but were rarely observed elsewhere. Most (33/43 [77%]) of the Ottawa *emm*3 strains had the *emm*3.53 allele, which differs from the *emm*3.2 allele by a single nucleotide change. Isolates with the *emm*3.2 allele are otherwise the most abundant *emm*3 strains in Ontario. We note that 1 isolate with the *emm*3.53 allele was found in Toronto in 2009, where it had not been observed in previous years, suggesting recent introduction. Subsequent studies will be required to determine whether the *emm*3.53 strain expands across Ontario and whether it has increased invasive potential.

## Discussion

In this large study of *emm* type distribution among GAS pharyngitis strains in Canada, we identified a similar pattern of *emm* type distribution as reported in previous surveys and also observed that strains of a relatively few *emm* types dominate. The most abundant *emm* types were similar to those reported in previous studies of GAS pharyngitis strains from North America and Europe (*8**,**9**,**12*).

**Figure 4:**
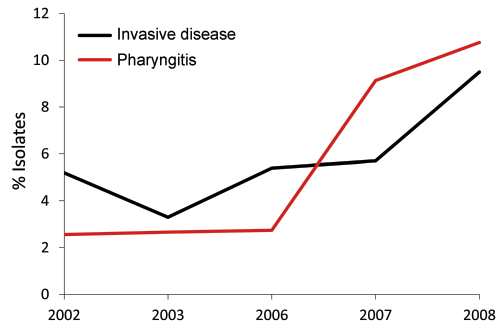
Frequency of *emm*89 strains among patients with group A *Streptococcus* pharyngitis and invasive disease, Ontario, Canada, 2002–2010, excluding 2004–2005. Black line indicates yearly frequency of *emm*89 among invasive disease isolates; red line indicates frequency of *emm*89 among pharyngeal isolates.

Of note, we found that *emm*89 strains have recently increased in frequency in Ontario. Specifically, over a 9-year period, *emm*89 strains increased 5-fold and in 2010 were the second most common *emm* type in the Toronto sample. The increase in *emm*89 strains among pharyngeal isolates paralleled an increase in the frequency of *emm*89 strains among invasive GAS isolates from 2003 through 2010 (Figure 4). This finding suggests that a marked expansion of *emm*89 strains has occurred in Ontario. Regional outbreaks of *emm*89 strains have been documented previously, including a clonal epidemic that occurred in northern Italy (*22*). Surveillance conducted by CDC also has reported similar increases in *emm*89 strains among invasive infections in New York and Maryland during 2007–2009, and *emm*89 was among the top 5 invasive serotypes collected by CDC in 1998, 2001–2003, and 2007–2009 (*23*). Thus, *emm*89 strains may commonly contribute to local epidemics of pharyngitis and invasive disease.

Although the fact that the frequency distribution of GAS pharyngitis *emm* types varies across localities separated by large distances has been well described, we have shown that *emm* type distributions also can vary over relatively small distances. This finding expands knowledge of GAS epidemiology. Thus, GAS pharyngitis strains circulating in Ontario are a collection of distinct populations, apparently characterized by relatively limited transmission between distant locations. Much of our understanding of GAS epidemiology has been based on the characterization of strains causing local outbreaks of invasive disease and large surveillance networks encompassing geographically expansive catchment regions. This circumstance has led to the belief that GAS exists mostly as large, homogeneous populations. Our findings suggest that GAS populations are much more complex. This conclusion is supported by our previous genomewide analysis of invasive *emm*3 isolates from Ontario, which found that genetic distance and geographic proximity were strongly correlated and that groups of clonally related isolates were frequently limited to discrete geographic locations (*24*).

Our longitudinal analysis of *emm* types in Toronto indicated that several *emm* types (including *emm*1, *emm*2, *emm*3, and *emm*77) varied substantially in annual frequency, which suggests features of epidemic behavior. Comparison of yearly frequencies of *emm* types in *emm3* isolates from patients with pharyngitis and invasive disease showed a nearly superimposable pattern, with coincident peaks of infection occurring in 2006 (Figure 3). This finding is consistent with a model in which many invasive GAS strains originate from the local pharyngitis strains and that cyclical outbreaks of invasive infection coincide or follow recent outbreaks of pharyngitis infections. A similar conclusion was reported by Hoe et al. (*25*), whose analysis of pharyngitis and invasive isolates from Finland showed that a novel streptococcal inhibitor of complement (*sic*) alleles first appeared in local pharyngitis strains before their appearance in invasive isolates. Furthermore, a GAS clone responsible for a local outbreak of invasive disease in Minnesota was common among pharyngeal isolates from school-aged children living in the outbreak area (*26*). Additional investigation into the genetic relationship between pharyngitis and invasive disease strains conducted at the full-genome level may provide useful information about the molecular events that contribute to invasive GAS.

The large size and longitudinal nature of our strain sample enabled us to obtain extensive information about the level of *emm* allelic variation and the rate of emergence of new *emm* alleles in Ontario. Previous experiments conducted by Dale et al. on 3 serotypes included in the 26-valent GAS vaccine found that slight allelic variants had little influence on bactericidal killing activity during in vitro assays, leading the researchers to conclude that variant subtypes might not affect vaccine efficacy (*27*). However, this finding contrasts with several other reports that observed a variable response to allelic variants (*28**,**29*). Whether the findings of Dale et al. are applicable to all 26 serotypes included in the vaccine is unknown (*27*). Despite its potential, albeit unproven, relevance to GAS vaccine design, we have a relatively limited understanding about this subject. We observed extensive *emm* allelic variation in Ontario, with most common *emm* types possessing >6 different alleles. We also found that strong selective pressure was driving the emergence of new M-protein variants, with all but one of the new alleles encoding amino acid substitutions. The observed ratio of synonymous to nonsynonymous nucleotide substitutions indicates that allelic variation most likely is shaped by selective pressure, perhaps immune mediated. Previous investigators have also reported that the N-terminal regions of M proteins possess functional domains in addition to opsonic epitopes that might constrain the amount of variability within an M type (*30**–**33*). Whether allelic variation may eventually result in escape mutants in a population with high levels of immunity, resulting from administration of an M-protein–based vaccine, is not known but should be considered. We believe this allelic variation might pose a challenge to GAS vaccine designs that rely on recombinant portions of the M-protein amino-terminus.

GAS M-protein serotypes are often regarded as genetically homogeneous populations composed of a single or relatively few clones. The remarkable level of *emm* type allelic diversity we observed in Ontario contrasts with this view. We found extensive diversity not only in the distribution of GAS serotypes but also on the allelic level and between geographic locations separated by short distances. Our recent genomewide analysis of invasive M3 isolates in Ontario revealed a strikingly complex genetic structure (*24*). Given the relationship between these populations, GAS pharyngeal isolates probably harbor an additional layer of genetic diversity that remains to be elucidated through whole-genome sequencing of a population of pharyngeal isolates.
